# Characterization of the MicroRNA Cargo of Extracellular Vesicles Isolated from a Pulmonary Tumor-Draining Vein Identifies miR-203a-3p as a Relapse Biomarker for Resected Non-Small Cell Lung Cancer

**DOI:** 10.3390/ijms23137138

**Published:** 2022-06-27

**Authors:** Bing Han, Laureano Molins, Yangyi He, Nuria Viñolas, David Sánchez-Lorente, Marc Boada, Angela Guirao, Tania Díaz, Daniel Martinez, Jose Ramirez, Jorge Moisés, Melissa Acosta-Plasencia, Mariano Monzo, Ramón M. Marrades, Alfons Navarro

**Affiliations:** 1 Molecular Oncology and Embryology Laboratory, Department of Surgery and Medical Specializations, Human Anatomy Unit, Faculty of Medicine and Health Sciences, Universitat de Barcelona (UB), c. Casanova 143, 08036 Barcelona, Spain; bhanhanx7@alumnes.ub.edu (B.H.); yangyihe@ub.edu (Y.H.); tdiaz@ub.edu (T.D.); macostapl@ub.edu (M.A.-P.); mmonzo@ub.edu (M.M.); 2 Department of Thoracic Surgery, Hospital Clínic de Barcelona, University of Barcelona, 08036 Barcelona, Spain; lmolins@clinic.cat (L.M.); dsanche1@clinic.cat (D.S.-L.); mboada@clinic.cat (M.B.); guirao@clinic.cat (A.G.); 3 Thoracic Oncology Unit, Hospital Clinic, 08036 Barcelona, Spain; nvinolas@clinic.cat (N.V.); dmartin1@clinic.cat (D.M.); jramirez@clinic.cat (J.R.); marrades@clinic.cat (R.M.M.); 4 Institut d’Investigacions Biomèdiques August Pi i Sunyer (IDIBAPS), c. Villarroel, 170, 08036 Barcelona, Spain; jrmoises@clinic.cat; 5 School of Basic Medical Sciences, Chengdu University, Chengdu 610106, China; 6 Department of Medical Oncology, Institut Clínic de Malalties Hemato-Oncològiques (ICMHO), Hospital Clínic de Barcelona, University of Barcelona, 08036 Barcelona, Spain; 7 Department of Pathology, Hospital Clínic de Barcelona, University of Barcelona, 08036 Barcelona, Spain; 8 Centro de Investigación Biomédica en Red de Enfermedades Respiratorias (CIBERES), Instituto de Salud Carlos III, 28029 Madrid, Spain; 9 Department of Pneumology, Institut Clínic Respiratori (ICR), Hospital Clínic de Barcelona, University of Barcelona, 08036 Barcelona, Spain

**Keywords:** lung cancer, NSCLC, tumor-draining vein, exosomes, extracellular vesicles, relapse biomarker, miRNAs, miR-203a-3p

## Abstract

In resected non-small cell lung cancer (NSCLC), post-surgical recurrence occurs in around 40% of patients, highlighting the necessity to identify relapse biomarkers. An analysis of the extracellular vesicle (EV) cargo from a pulmonary tumor-draining vein (TDV) can grant biomarker identification. We studied the pulmonary TDV EV-miRNAome to identify relapse biomarkers in a two-phase study (screening and validation). In the screening phase, a 17-miRNA relapse signature was identified in 18 selected patients by small RNAseq. The most expressed miRNA from the signature (EV-miR-203a-3p) was chosen for further validation. Pulmonary TDV EV-miR-203a-3p was studied by qRT-PCR in a validation cohort of 70 patients, where it was found to be upregulated in relapsed patients (*p* = 0.0194) and in patients with cancer spread to nearby lymph nodes (N+ patients) (*p* = 0.0396). The ROC curve analysis showed that TDV EV-miR-203a-3p was able to predict relapses with a sensitivity of 88% (AUC: 0.67; *p* = 0.022). Moreover, patients with high TDV EV-miR-203a-3p had a shorter time to relapse than patients with low levels (43.6 vs. 97.6 months; *p* = 0.00703). The multivariate analysis showed that EV-miR-203a-3p was an independent, predictive and prognostic post-surgical relapse biomarker. In conclusion, pulmonary TDV EV-miR-203a-3p is a promising new relapse biomarker for resected NSCLC patients.

## 1. Introduction

Non-small cell lung cancer (NSCLC) is the most frequent type of lung cancer. Despite regional differences in incidence and mortality, NSCLC continues to be the most frequent cause of cancer-related death worldwide [1]. According to the American Lung Association, in a 2021 report, high mortality is mainly explained by the fact that only 24% of cases are diagnosed at an early stage, where the survival rate is much higher than in advanced stages (5-year survival rate of 60% vs. 6%). In early-stage patients, the preferential treatment is the surgical resection of the tumor, which is considered the most effective treatment, followed by a wait-and-watch strategy in stage I patients or by the administration of an adjuvant treatment in stage II patients. Despite the effectiveness of surgical treatment, around 40% of patients suffer from tumor recurrence within the first 5 years of surgery [2]. There is a necessity to identify patients with a high risk of relapse after surgery to improve treatment and surveillance plans for these patients [3]. However, there are few reliable biomarkers to predict post-operative relapses. One of the clinical predictors of post-operative recurrence and shorter disease-free survival is the positive detection of tumoral cells in the regional lymph nodes (N status) obtained during the surgery. The patients with no regional lymph node metastasis (N0) have better post-operative survival (5-year disease-free survival rate of 60%) than patients with lymph node metastasis (N+; 5-year disease-free survival rate of 44% for N1 or 29% for N2 patients). Moreover, some molecular markers (i.e., KRAS or Ki-67); individual microRNAs (miRNAs), such as miR-34a [4] or miR-141 [5]; or even miRNA signatures, including let-7a, miR-7, miR-21, miR-155, miR-210 and miR-221 [6], have been indicated as potential relapse biomarkers, but their clinical use has not been consolidated [2]. Therefore, there is a need for the identification of new post-operative relapse biomarkers and their clinical consolidation. In this line, liquid biopsy, which is based on detecting tumor-related products in body fluids, has received much attention as a source of relapse biomarkers during the last years [7]. Several tumor-derived products have been studied in body fluids from NSCLC patients, including circulating tumor cells (CTCs) [8,9,10], circulating DNA [11,12], circulating miRNAs [13,14,15] or extracellular vesicles (EVs) [16] and their cargo [17,18].

Small EVs are defined as non-replicative particles of less than 150 nm in size delimited by a lipid bilayer released by the cells with cell-to-cell communication functions [19]. Different types of particles can be included in this definition, with exosomes being the most relevant ones. Exosomes are produced in a fine regulated process by multivesicular bodies and are released with cell-to-cell communicative functions and with a relevant role in either the regulation of the local tumor microenvironment or in participating in the metastasis process through interactions with CTCs and/or preparing the pre-metastatic niche [20]. Exosome functions are performed mainly by fusion with the target cell and releasing cytoplasmic cargo that is enriched in small non-coding RNAs, mainly miRNAs [21]. Therefore, the study of the cargo can be a good way to identify relapse biomarkers.

In surgical patients, the obtention of blood from the tumor-draining vein (TDV) has been shown as an exceptional opportunity to study blood enriched in products released by the tumor. In this regard, several studies have demonstrated that TDV is enriched in circulating tumor cells [22,23] and also in tumor-derived EVs [24]. In addition, in both cases, their analysis in TDV has been demonstrated to have a larger prognostic impact than in peripheral veins either in NSCLC [24,25,26] or in other tumors [27,28,29]. To date, however, no reports have examined the small EV-miRNAome cargo in TDV and their potential role as post-operative relapse biomarkers in NSCLC.

In the present study, we performed a miRNA screening in small EVs purified from pulmonary TDV from 9 relapsed and 9 non-relapsed NSCLC patients to identify post-operative relapse biomarkers, which were validated in a cohort of 70 resected NSCLC patients.

## 2. Results

### 2.1. Screening Phase

#### 2.1.1. Patients

The screening cohort included 18 patients: 9 relapsed and 9 non-relapsed. A total of 2 patients were stage I, 13 were stage II and 3 were stage III. A total of 12 (66.7%) patients received adjuvant treatment after surgery (8 stages II and 4 stages III). The median follow-up time was 66.07 months (IQR: 57.97–82.93). The main clinical characteristics are summarized in Table 1.

#### 2.1.2. miRNA Profile of EV Purified from Pulmonary TDV

**Figure 1:**
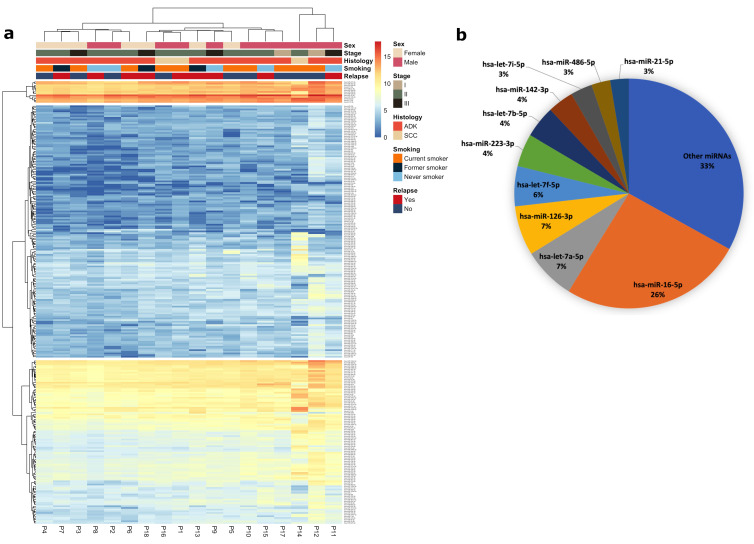
Analysis of the miRNA profile of pulmonary TDV EVs from NSCLC patients: (**a**) Heat map showing the expression (TPM) of the 262 miRNAs detected in the EV from the pulmonary TDV from NSCLC patients. (**b**) Pie chart of the top ten most abundant EV miRNAs identified in pulmonary TDV from NSCLC patients.

From the 2578 miRNAs listed in miRbase, we detected 1165 in the EVs from pulmonary TDV. However, only 262 miRNAs had an expression of a minimum of 10 tags per million mapped reads (TPM) in at least three patients. Using these 262 miRNAs, we drew a heat map (Figure 1a), where the miRNA expression pattern in the different samples and the main patient characteristics (sex, stage, histology, smoking history and relapse after surgery) can be observed. The hierarchical cluster for miRNAs shows three main clusters, which classify the miRNAs as high, medium or low expressed miRNAs. Moreover, three main clusters were also observed for patients, but no significant clustering was observed according to the main clinical characteristics (chi-squared analysis showed a *p* > 0.05).

Of note, only 127 miRNAs were expressed in all samples with at least 10 TPM per sample (Table S1). The most abundant EV miRNA in our samples was miR-16-5p, which represents 26% of all obtained raw counts. The top ten abundant miRNAs are shown in Figure 1b and account together for 67% of the total raw counts obtained. All of them are clustered together in the high expression cluster, seen at the top of the heatmap.

#### 2.1.3. Identification of miRNAs Associated with Relapse

**Figure 2:**
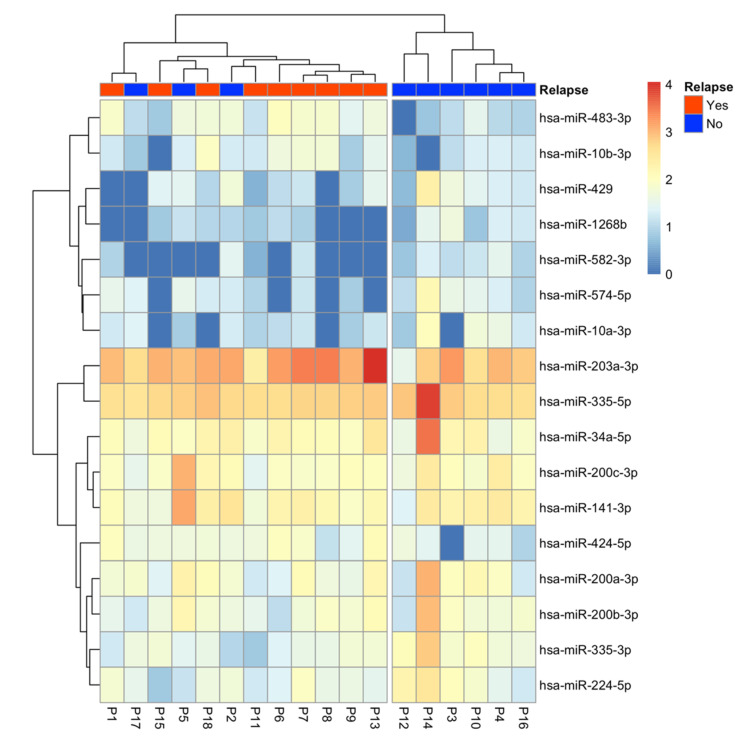
Hierarchical cluster analysis showing the expression of the 17 differentially expressed miRNAs between relapsed and non-relapsed patients. Log10 TMM was drawn, and Pearson correlation was used as clustering distance.

The supervised analysis allowed us to identify 17 miRNAs differentially expressed between relapsed and non-relapsed patients (Table 2). A total of 4 miRNAs were upregulated in relapsed patients, whereas 13 miRNAs were downregulated. The hierarchical cluster analysis of these 17 miRNAs showed two well-defined clusters. The left cluster included three non-relapsed and the nine relapsed patients, whereas the right cluster included the six remaining non-relapsed patients (Fisher’s exact test: *p* = 0.0090; Figure 2).

#### 2.1.4. Identification of EV-miR-203a-3p as a Candidate Relapse Biomarker

**Figure 3:**
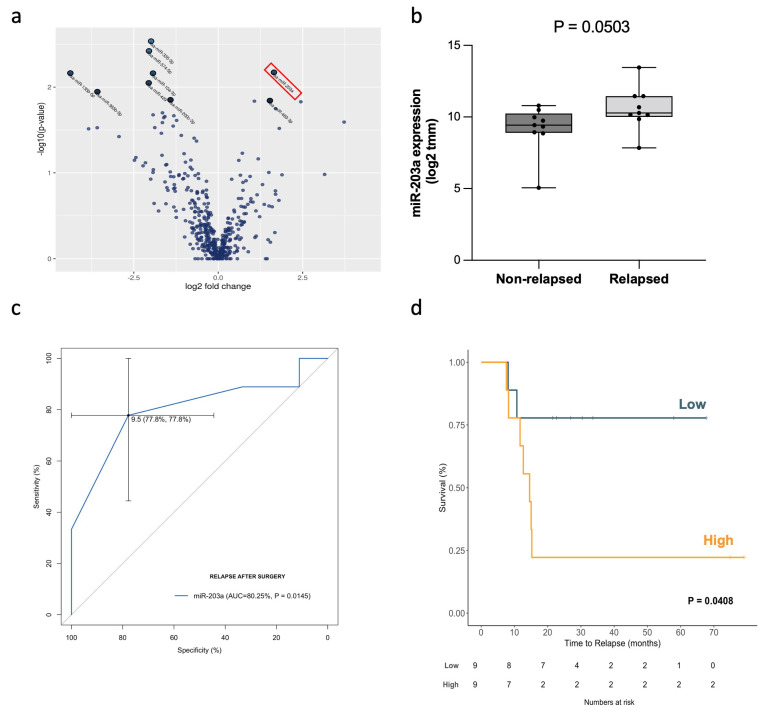
TDV EV-miR-203a-3p was identified as a candidate relapse biomarker: (**a**) Volcano plot of miRNAs differentially expressed in pulmonary TDV EVs from relapsed and non-relapsed NSCLC patients. EV-miR-203a-3p (highlighted with a red box) was the most upregulated miRNA in relapsed patients. (**b**) Boxplot showing TDV EV-miR-203a-3p levels (log2 TMM) in non-relapsed and relapsed patients from the screening cohort. The *p*-value shown has been calculated using the Mann–Whitney U test. (**c**) ROC curve analysis of pulmonary TDV EV-miR-203a-3p, predicting relapse after surgery in the screening cohort of NSCLC patients. (**d**) Kaplan–Meier survival analysis of pulmonary TDV EV-miR-203a-3p and TTR in the screening cohort of NSCLC patients.

To identify the most promising relapse biomarker from the list of 17 miRNAs identified in the small RNAseq analysis, we prioritized the study of the most significant upregulated miRNAs in the relapsed group. As observed in the volcano plot analysis (Figure 3a), the 2 most upregulated miRNAs were miR-203a-3p and miR-483-3p. We carefully evaluated the number of reads obtained for each miRNA (to avoid selecting significant miRNAs with very few observed reads). Whereas miR-203a-3p had a total of 4490 reads (mean: 249 reads per sample; range: 84–1612), miR-483 had only 86 reads in total (mean: 4.7 reads per sample; range: 0–86). Therefore, we decided to focus on miR-203a-3p, whose expression in relapse and non-relapsed patients is summarized in Figure 3b.

To analyze its potential as a relapse biomarker and its impact on the time to relapse (TTR), we performed an ROC curve and a Kaplan–Meier analysis, respectively. The area under the curve (AUC) value, according to pulmonary TDV EV-miR-203a-3p, was 0.80 (95% confidence interval [CI], 0.58–1.0; *p* = 0.0145) (Figure 3c).

To analyze whether TDV EV-miR-203a-3p impacted TTR, a Cox univariate analysis was performed and showed that TDV EV-miR-203a-3p, as a continuous variable, impacted the TTR in the screening cohort (HR: 2.268; 95%CI: 1.082–4.755; *p* = 0.0302). The Kaplan–Meier analysis (using median expression as a cutoff point) showed that high TDV EV-miR-203a-3p patients had shorter TTR (27 vs. 54.7 months; *p* = 0.0408; Figure 3d) than those with low expression.

### 2.2. Validation Phase

#### 2.2.1. Characterization of Isolated EVs

**Figure 4:**
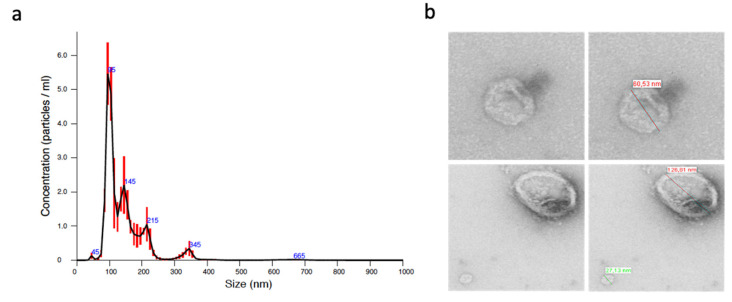
Characterization of EVs obtained by ultracentrifugation by (**a**) nanoparticle tracking analysis and (**b**) transmission electron microscopy. In the top right picture, a 60.53 nm vesicle is shown, and in the lower right picture, 126.81 nm and 27.13 nm vesicles are shown.

Prior to purifying EVs from all samples from the validation cohort, we verified by different methods that we were correctly purifying EVs. Nanoparticle tracking analysis (NTA) showed that most of the particles that were obtained had less than 150 nm, as shown in Figure 1a. Moreover, the morphological analysis by transmission electron microscopy (TEM) showed the presence of round 27–126 nm vesicles (Figure 4b).

#### 2.2.2. Exploratory Analysis of the Expression of miR-203a-3p in Tissue and Peripheral Blood

**Figure 5:**
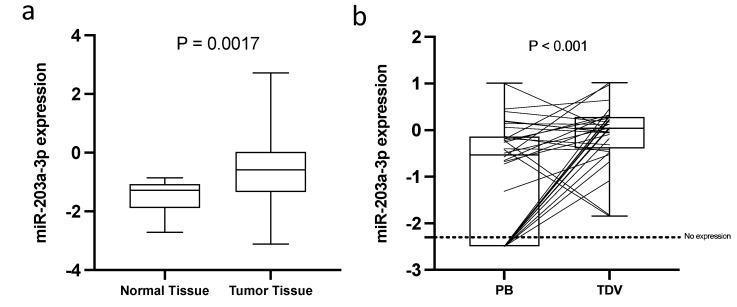
Exploratory analysis of the expression of miR-203a-3p in tissue and EV obtained from PB: (**a**) Boxplot showing the expression of miR-203a-3p in 20 normal tissues and in 30 tumor tissues. (**b**) Boxplot combined with before and after plot showing the expression of miR-203a-3p in 32 paired samples of EV from PB or TDV.

We explored the expression pattern of miR-203a-3p in tissue (normal [*n* = 20] vs. tumor tissue [*n* = 30]) and peripheral blood (PB [*n* = 32] vs. TDV blood). We observed that miR-203a-3p was significantly upregulated in tumor tissue in comparison to normal tissue (*p* = 0.0017; Figure 5a). Additionally, using TCGA data (obtained from the MIR-TV web page), we validated that, in both, LUAD (for adenocarcinoma patients) and LUSC (for squamous cell carcinoma patients) TCGA cohort miR-203a-3p was significantly upregulated in tumors vs. normal tissue (*p* < 0.001; Figure S1).

Next, we analyzed miR-203a-3p expression in EV purified from PB in comparison to EV from TDV. The expression of miR-203a-3p was significantly higher in TDV than it was in peripheral vein EV. It is important to highlight that, in PV, the expression of miR-203a-3p was not detected in 34% of the patients (Figure 5b).

#### 2.2.3. Characteristics of the Patients from the Validation Cohort

The validation cohort included 70 patients (Table 3). A total of 38 patients were stage I, 25 were stage II and 7 were stage III. A total of 24 patients received adjuvant chemotherapy (18 were stage II and 6 were stage III). The median follow-up time was 54.23 months (IQR: 39.20–63.97). A total of 25 patients (35.7%) relapsed after surgery, and 16 of them had distant metastases.

#### 2.2.4. TDV EV-miR-203a-3p Expression Is Associated with N and Relapse

**Figure 6:**
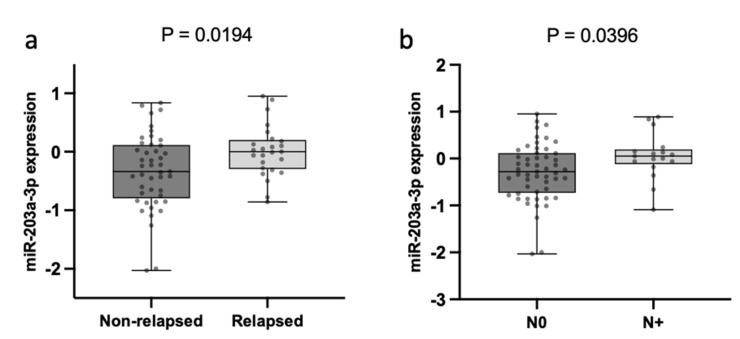
TDV EV-miR-203a-3p expression and clinical variables: (**a**) Boxplot showing EV-miR-203a-3p levels in non-relapsed and relapsed patients. (**b**) Boxplot showing expression levels in N0 vs. N+ patients.

The correlation of TDV EV-miR-203a-3p expression with the main clinicopathological characteristics only showed a significant association with relapse after surgery and N status. Relapsed patients had higher levels of TDV EV-miR-203a-3p than non-relapsed patients (*p* = 0.0194; Figure 6a). Moreover, N+ patients had higher levels of TDV EV-miR-203a-3p than N0 patients (*p* = 0.0396; Figure 6b).

#### 2.2.5. EV-miR-203a-3p Levels Validated as Relapse Biomarkers in the Validation Cohort

**Figure 7:**
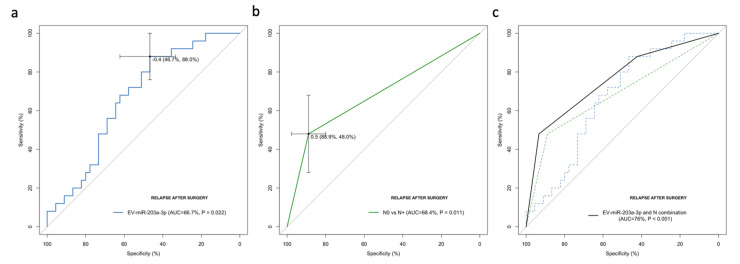
ROC curve analysis for the prediction of post-surgical relapse in NSCLC patients according to (**a**) TDV EV-miR-203a-3p, (**b**) N status and (**c**) the combination of TDV EV-miR-203a-3p (high vs. low) and N (N0 vs. N+).

ROC curves were generated to investigate EV-miR-203a-3p as a predictive biomarker of relapse after surgery. The AUC value, according to pulmonary TDV EV-miR-203a-3p, was 0.67 (95% CI: 0.540–0.793; *p* = 0.022), with a sensitivity of 88% and a specificity of 46.7% in distinguishing patients who relapse after surgery in the best threshold (−0.4) (Figure 7a). Using −0.4 as a threshold, the negative predictive value was 87.5%, and the positive predictive value was 47.8%.

To compare the impact of EV-miR-203a-3p with other clinical factors, N and disease stages were included in the analysis. N0 vs. N+ showed an AUC of 0.684 (95%CI: 0.546–0.823; *p* = 0.011; Figure 7b), and the disease stage showed an AUC of 0.692 (95%CI: 0.559–0.824; *p* = 0.008). To verify that no other confounding parameters could have affected these results, we performed a binary logistic regression multivariate analysis for relapsing to verify the independent impact of EV-miR-203a-3p to predict relapses after curative surgery. High EV-miR-203a-3p (HR: 4.728; 95%CI: 1.168–19.143; *p* = 0.029) and N (HR: 5.588; 95%CI: 1.576–19.813; *p* = 0.008) emerged as independent markers of relapse.

Of note, when we combined TDV EV-miR-203a-3p and N, an improved AUC of 0.760 (95%CI: 0.639–0.882) was obtained (Figure 7c).

#### 2.2.6. EV-miR-203a-3p Levels Impact TTR

**Figure 8:**
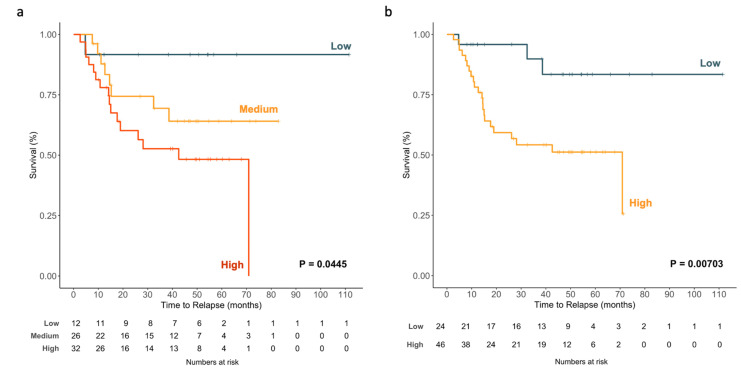
Kaplan–Meier survival analysis of TDV EV-miR-203a-3p and TTR among patients with NSCLC: (**a**) Patients were classified according to TDV EV-miR-203a-3p levels as low, medium or high. (**b**) Patients were classified as low or high according to the cutoff identified in the ROC curve analysis.

EV-miR-203a-3p expression was subjected to univariate Cox regression analysis with TTR as the dependent variable, which showed that EV-miR-203a-3p, as a continuous variable, impacted the TTR (HR: 2.384; 95%CI: 1.142–4.977; *p* = 0.021). A graphical representation of the impact as a continuous variable on TTR can be observed in Figure 8a, where patients are classified according to TDV EV-miR-203a-3p expression as being part of the low, medium or high group (*p* = 0.0445).

Using the best cutoff point identified using the ROC analysis, 24 patients (34.3%) were classified as having low expression, and 46 (65.7%) were classified as having high expression. High TDV EV-miR-203a-3p patients had shorter TTR (43.6 vs. 97.6 months; *p* = 0.00703; Figure 8b) than those with low expression.

Finally, we analyzed whether the impact observed in the TTR of TDV EV-miR-203a-3p could be observed in EV from PB. As can be observed in Figure S2, we could not find a significant difference in the TTR (*p* = 0.603) between patients with high or low expression of PB EV-miR-203a-3p, whereas a trend of significance was observed in TDV (*p* = 0.0612) for the same paired 32 samples.

#### 2.2.7. Cox Modeling of Relapse

In the multivariate Cox analyses (Table 4), TDV EV-miR-203a-3p levels emerged as an independent risk factor for the TTR (HR: 2.442; 95%CI: 1.126–5.294; *p* = 0.024). Stage I was also an independent marker of the TTR (HR: 0.174; 95%CI: 0.056–0.543; *p* = 0.003).

#### 2.2.8. Impact on the TTR of the Combination of N and EV-miR-203a-3p Levels

**Figure 9:**
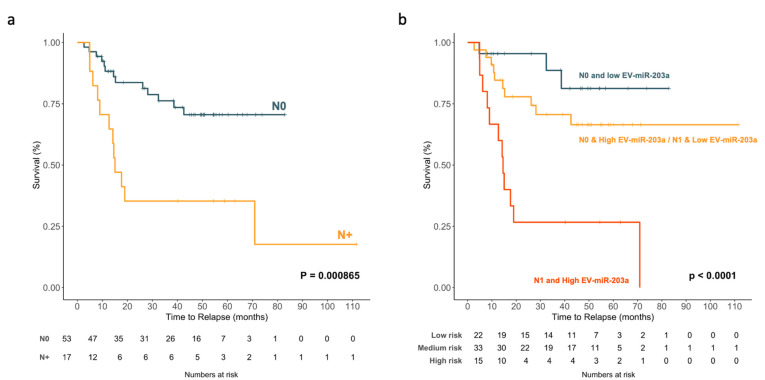
Kaplan–Meier survival analysis for TTR of (**a**) N0 vs. N+ and (**b**) the combination of N and TDV EV-miR-203a-3p levels among patients with NSCLC.

Finally, we decided to explore the potential effects on the TTR of combining N status and TDV EV-miR-203a-3p. When we analyzed the impact of N status on TTR, we observed that the 17 (24.3%) N+ patients had a shorter TTR than the 53 (75.7%) N0 patients (39.6 vs. 64.6 months; *p* = 0.000865, Figure 9a).

We combined N status and TDV EV-miR-203a-3p to obtain three different groups: patients with N0 and low EV-miR-203a-3p (low-risk group); patients with N0/high EV-miR-203a-3p or N1/low EV miR-203a-3p; and patients with N1/high EV-miR-203a-3p (high-risk group). The high-risk group had the shortest TTR (27.32 months, *p* < 0.0001; Figure 9b).

## 3. Discussion

miRNAs can represent a large percentage (around 40%) of EV RNA cargo [21], and for this reason, they have been extensively studied as potential biomarkers in several tumors, including NSCLC, where a huge number of EV miRNAs have been identified as diagnostic and even as prognostic biomarkers (reviewed in [30]). However, to our knowledge, all the previous studies have been performed in EVs purified from PB, where tumor-derived EVs are highly diluted. In the present study, we focused on the analysis of the miRNA cargo from EVs purified from pulmonary TDV, where we have previously showed that there is enrichment in small exosomes (30–50 nm) in comparison with paired PB, and their levels had a better correlation with the clinicopathological characteristics of the patient than in PB [24]. The miRNA profile of pulmonary TDV EVs shows the top ten most abundant EV-miRNAs that some have previously identified as diagnostic biomarkers in PB, since the detection of their expression is associated with the presence of lung cancer. EV-miR-21-5p has been used to discriminate patients with lung adenocarcinoma from healthy subjects [31]. EV-miR-21 overexpression has been included in a mathematical model together with two additional miRNAs to be used for the early detection of NSCLC [32]. Moreover, miR-21 has previously been described as one of the highest EV-miRNAs in NSCLC patients [33]. EV-miR-let-7b-5p, let-7e-5p and miR-486-5p have also been identified as potential diagnostic biomarkers of stage I NSCLC patients [34]. The identification of these miRNAs in our top ten supports the hypothesis that the TDV is enriched in tumor-derived EVs.

We designed the study to identify miRNAs related to post-operative recurrence, and therefore, for the screening phase, we retrospectively selected nine patients who relapsed after surgery and nine patients in whom, after a follow up of at least 2 years, no relapse was detected. The supervised analysis allowed us to identify a relapse-related miRNA signature, including the upregulation of miR-10b-3p, miR-203a-3p, miR-424-5p and miR-483-3p and the downregulation of miR-10a-3p, miR-34a-5p, miR-1268b, miR-141-3p, miR-200a-3p, miR-200b-3p, miR-200c-3p, miR-224-5p, miR-335-3p, miR-335-5p, miR-429, miR-574-5p and miR-582-3p in relapsed patients. The role of these miRNAs in relapse are discussed in the following lines.

miR-10b is highly expressed in metastatic breast cancer cell lines and is actively secreted in the medium via EVs to promote cell invasion [35]. Moreover, it was described as upregulated in EVs from NSCLC adenocarcinoma patients, and the analysis of the prognostic impact using TCGA tissue data showed that high levels were correlated with shorter overall survival [36]. Aggressive prostate cancer releases miR-424 containing EVs [37]. High levels of EV-miR-483-3p have been associated with shorter overall survival after pancreatic resection in pancreatic ductal adenocarcinoma [38].

EV-miR-10a has been described as a negative regulator of the migration of human lung fibroblasts in a colorectal cancer study. The authors have suggested that it can be involved in the modulation of the lung microenvironment during the metastasis process [39]. We can speculate that a similar role can be played in the modulation of the microenvironment to enhance the dissemination of lung cancer cells. miR-34a-5p has been described by our group as a tumor suppressor prognostic biomarker in NSCLC [4]. We showed that patients with low levels in tumor tissue had a shorter time to relapse. Moreover, Zhao et al. described that circulating miR-34a family low expression correlated with poor DFS and OS in NSCLC patients [40]. miR-1268b has been described as a diagnostic biomarker in serum samples of NSCLC [41]. In hepatocellular carcinoma, low expression levels of miR-1268a have been correlated with poor overall survival and poor tumor recurrence-free survival [42].

Additionally, in our relapse signature, we observed several members of the miR-200 family, including miR-141-3p, miR-200a-3p, miR-200b-3p, miR-200c-3p and miR-429. These miRNAs have been extensively studied by their regulation of the epithelial–mesenchymal process and therefore by the regulation of the metastasis process [43]. Their expression in liquid biopsies has been described in cell-free plasma/serum [44], in sputum [45] and in EVs from pleural fluid [46], where they have been used as diagnostic biomarkers, since they have been observed to be upregulated in lung cancer samples in comparison to controls. However, to our knowledge, no studies have reported regarding their role as relapse biomarkers in EVs from NSCLC patients.

miR-335-5p downregulation in NSCLC tumor tissue [47] is associated with lymph node metastasis through the regulation of ROCK1 [48]. Moreover, patients with lower miR-335-5p experienced poor overall survival [49].

All these results are in line with the role as a relapse biomarker of our TDV EV-miRNA signature. However, controversial functions have been described for three miRNAs (miR-574-5p, miR-582-3p and miR-224-5p) included in the signature, not supporting their potential role as relapse biomarkers. MiR-574-5p has been previously observed that it was upregulated in lung cancer patients vs. controls and that it decreases their expression in PB after surgical resection of the tumor [50]. miR-582-3p has been described as upregulated in hypoxic conditions and is secreted in EVs to promote tumorigenesis [51], and EV-miR-224-5p has been shown to promote tumorigenesis in NSCLC [52]. However, some of these results have been only observed in cell lines, and regardless, their expression has been analyzed in TDV.

We decided to focus our attention on the miRNAs that were most upregulated in relapsed patients, and, specifically we chose miR-203a-3p for further study and validation. In the screening cohort, higher levels of EV-miR-203a-3p acted as a relapse predictor and as a poor TTR biomarker. We validated these results in the validation cohort, where we observed that significantly higher EV-miR-203a-3p levels were found in relapsed patients and in patients with lymph node metastasis (N+). Moreover, the role as a relapse predictor biomarker, although it was validated, was not so remarkable as it was in the screening cohort. However, when compared with the prediction capacity of the disease stage or N, we observed that the prediction capacity was comparable and independent according to the multivariate analysis. Of note, the combination of N and EV-miR-203a-3p had a greater prediction capacity than both factors alone. Moreover, we would like to highlight that relapsing after surgery is a time-dependent variable, and when we account for the time in the TTR analysis, the obtained results are more outstanding than its relapse prediction capacity. Then, when we validated the role of EV-miR-203a-3p as a prognostic biomarker for TTR, we observed that, as continuous variable, EV-miR-203a-3p impacted relapse, where higher levels were associated with a shorter TTR. Moreover, the Kaplan–Meier analysis confirmed that patients with higher levels had a shorter TTR, and it emerged as an independent prognostic factor in the multivariate analysis together with the disease stage. Despite TDV EV-miR-203a-3p acting as a relapse biomarker, it is clearly a better TTR biomarker. Since we previously observed that the combination with N status improved the capacity of relapse prediction, we also decided to combine EV-miR-203a-3p and N for the TTR analysis, resulting in observing a summatory effect, where the patients with N+ and high EV-miR-203a-3p had the worst outcomes.

miR-203a-3p was identified as more enriched in EVs from the pleural fluid of NSCLC patients compared to benign lesions, such as tuberculosis [46,53]. Other authors have shown that EV-miR-203-3p levels are able to discern between NSCLC and small cell lung cancer [54] and also have been described as a biomarker for adenocarcinoma [33]. The role of miR-203a-3p as a tumor suppressor or oncogene seems to depend on if it is studied in the tissue or in EVs. In lung cancer tissues, miR-203a-3p downregulation has been associated with advanced TNM stages, lymph node metastasis and poor prognosis in an Asian cohorts [55]. However, in our cohort, miR-203a-3p was upregulated in tumor tissue in comparison with normal tissue, indicating that there may be differences in expression according to cohort characteristics, since the analysis of TCGA data supports our results regarding tumor tissue. In colorectal cancer tissues, where low miR-203a-3p expression is correlated with shorter overall survival, the analysis of EV-miR-203a-3p in serum samples showed the contrary. High EV-miR-203a-3p levels were associated with worse outcomes and have been related to metastasis via inducing tumor-associated macrophages [56]. The authors have speculated about the site-dependent function of miR-203a-3p, which seems to act as a tumor suppressor in the primary cancer tissue in cancer cells, while acting as an oncogene in EVs acting over monocytes. This observation is in line with our results, but the real target identification of EV-miR-203a-3p deserves further study and functional validation. This is out of the scope of the present paper. However, we performed an exploratory analysis using miRPathDB v2.0 to identify potential pathways regulated by miR-203a-3p, and from the 1960 pathways that were identified (Table S2), we highlighted the observations of the top 50 of pathways related to metabolism regulation, DNA transcription or cell migration between others.

We are aware that the present manuscript has several limitations. This is a retrospective study with a relatively small cohort that lacks independent validation in an external cohort. Access to an independent cohort of samples from TDV is challenging, making the validation of the results difficult, as most of the groups are working with peripheral veins, and there are no available data on public repositories for in silico validation. In addition, another limitation of the study is the fact that, based on the origin of the blood, we cannot extend our results to advanced or to non-surgical early-stage NSCLC patients, and we are limited to surgical patients. However, it is important to clarify that the obtention of the TDV is safe, with no complications reported in our cohort of patients or in previous studies [25]. Another limitation that we need to account for is that there is not a standard and reliable method to purify EV yet. In the present work, we used in the validation cohort the ultracentrifugation method, which is one of the most used, but this method has some drawbacks, such as lower exosome quality due to vesicle damage, which in part is the explanation for why the purification method used for the obtention of the genomic data in the screening cohort was based on an EV precipitation technique. Moreover, with ultracentrifugation, we cannot separate the different populations of EVs, such as exosomes or microvesicles. It is difficult to identify the cell of origin related to our results, and we cannot assume that the genomic landscape of the metastasis is like that of the EVs that are analyzed. An EV subpopulation genomic analysis is more informative, as shown in [57], but is out of the scope of the present paper. However, we first need a standardization of the EV purification methodology to improve its clinical application.

In summary, in the present paper, for the first time, we analyzed the EV-miRNAome in pulmonary TDV, identifying a post-operative relapse miRNA signature, whose role in the metastasis process deserves further investigation as well as its potential use in other cancers. Moreover, we validated the prognostic impact of the most expressed miRNA of the signature, EV-miR-203a-3p, which is a promising TTR biomarker for surgical NSCLC patients and can be a useful tool to guide treatment decisions regarding adjuvant therapy. Further investigation in a prospective study is warranted to validate these findings and to decipher the functional relevance of pulmonary TDV EV-miR-203a-3p.

## 4. Materials and Methods

### 4.1. Study Design

This retrospective study is divided into 2 phases: the screening phase (*n* = 18 patients, Table 1) and the validation phase (*n* = 70 patients, Table 3). The aim of the screening phase was to identify EV miRNAs differentially expressed between relapsed and non-relapsed patients. In the validation phase, the prognostic impact of the identified EV miRNAs was validated in the whole cohort of patients.

### 4.2. Patient Samples

The study included 70 patients with pathological stages of I–IIIa who underwent complete surgical resection from 2011 to 2020 in the Hospital Clinic of Barcelona. Additional inclusion criteria were being older than 18 years; having stage I–II at diagnosis and being treated with surgery; being followed by thoracic oncology or by the Medical Oncology department at the Hospital Clinic of Barcelona for at least two years after surgery; and signing informed consent. Exclusion criteria were receiving neoadjuvant treatment before surgery; having simultaneous neoplasms; or dying due to postsurgical complications.

During surgery, 5 mL of blood was collected from the pulmonary TDV in an EDTA tube prior to tumor removal and pulmonary vein ligation [24]. Plasma was isolated by centrifugation (5000× *g* for 10 min) and was stored at −80 °C until processing. Additionally, peripheral blood, the tumor and adjacent normal tissue were also saved for most of the patients.

Written informed consent was obtained from each participant in accordance with the Declaration of Helsinki, and the study was approved by the Clinical Research Ethics Committee of the Hospital Clinic of Barcelona (project approval number HCB/2017/1052).

### 4.3. EV miRNA Profiling by Small RNAseq in the Screening Phase

The expression profile of EV miRNAs was determined by small RNAseq in a selected group of 18 patients (9 relapsed and 9 non-relapsed). The main clinical characteristics of the analyzed patients are showed in Table 1. The whole process, including EV isolation, RNA purification, RNA quality control, library preparation and sequencing, was performed by Exiqon—Qiagen Genomics Services (Hilden, Germany). Library preparation was performed using the QIAseq miRNA Library Prep kit. The analysis was performed in NextSeq500 (Illumina, San Diego, CA, USA).

### 4.4. Bioinformatic Analysis of Small RNAseq Data

Sequencing data were de-multiplexed, and FASTQ files for each sample were generated using bcl2fastq software (Illumina Inc., San Diego, CA, USA). FASTQ data were checked using the FastQC tool. Trimming and UMI correction were performed using Cutadapt (1.11) and an in-house script from Qiagen services. Read mapping was performed using Bowtie2 (2.2.2) taking into consideration that not more than one mismatch was allowed in the first 32 bases of the read and that no indels were allowed in mapping. The reference genome used in the analysis was GRCh37, and miRNAs were annotated using miRbase_20 as a reference. For normalization, the trimmed mean of M-values method based on log fold changes and absolute gene-wise changes in expression levels between samples (TMM normalization) was used. Differential expression analysis was performed on TMM normalized data using the Bioconductor EdgeR package in R. A volcano plot and a heat map were drawn with the differentially expressed genes identified. To generate a heat map of expression profiles in R, log10 TMM normalized quantifications were used.

### 4.5. EV Purification and Characterization in the Validation Phase

EVs from pulmonary TDV plasma were purified using our previous detailed methodology [58] by the ultracentrifugation technique. Briefly, EVs were isolated from 200 μL of plasma by ultracentrifugation in a Sorvall MX Plus Micro-Ultracentrifuge with an S140AT Rotor (Thermo Scientific, Waltham, MAS, USA). The characterization of the obtained EVs, with the aim of verifying their correct purification, was performed by transmission electron microscopy (TEM), nanoparticle tracking analysis (NTA) and Western blotting, as previously described [24]. Samples were observed using a TEM JEOL J1010 80 kV at the Electron Cryomicroscopy Unit of the University of Barcelona (CCiTUB, Barcelona, Spain). NTA was performed on a NanoSight NS300 in the ICTS “NANBIOSIS” (Biomaterial Processing and Nanostructuring Unit of the CIBER in Bioengineering, Biomaterials and Nanomedicine at Institut de Ciència de Materials de Barcelona, CSIC, Campus UAB, Bellaterra, Barcelona, Spain).

### 4.6. RNA Extraction and miRNA Expression Analysis in the Validation Phase

Once the EVs were purified, the supernatants were removed and resuspended with Qiazol, and RNA was extracted using the miRNeasy Mini Kit (Qiagen, Hilden, Germany), as previously performed [26]. RNA from tissue samples were purified using Trizol (ThermoFisher, Waltham, MAS, USA). Taqman microRNA assays (Life Technologies, Carlsbad, CA, USA) were used to quantify the expression of miR-203a (hsa-miR-203a-3p, 000507). Relative quantification was calculated using 2^−ΔΔCt^. miR-191 (002299) was used as an endogenous control.

### 4.7. Statistical Analysis

TTR was defined as the time from surgery to recurrence or the last follow-up. Optimal cutoffs for TTR for miR-203a-3p expression were obtained using the X-Tile program [59], and Kaplan–Meier curves were generated and compared by means of a log-rank test. All factors with *p* < 0.1 in the univariate analysis were included in the Cox multivariate regression analyses for TTR. *T*-tests or Mann–Whitney U tests were used for comparisons between two groups, and ANOVA was used for more than 2 groups. ROC curves were constructed and compared using the pROC package of R (The comprehensive R archive network; CRAN). All statistical analyses were performed using GraphPad Prism v9.3.1 and R v4.1.1.

## 5. Conclusions

In resected NSCLC patients, analyses of TDV EV miRNA cargo can provide accurate information on the likelihood of disease recurrence after surgery. Specifically, analyses of TDV EV-miR-203a-3p can be used as prognostic biomarkers for the identification of patients at high risk of relapse after surgery and can complement the information provided by main clinical factors.

## Figures and Tables

**Table 1:** Main clinical characteristics of screening cohort (*n* = 18) grouped by relapse status. The *p*-value was calculated using Fisher’s exact test or chi-squared, except for age, where a t-test was used. No significant differences were observed between the two groups of patients.

Characteristics	Subtypes	Non-Relapsed Patients *n* (%)	Relapsed Patients *n* (%)	*p*-Value
**Sex**	Male	6 (66.7)	5 (55.6)	
	Female	3 (33.3)	4 (44.4)	1.0
**Age, years**	Mean age (range)	61 (33–79)	64 (36–80)	0.287
	≤65	7 (77.8)	4 (44.4)	
	>65	2 (22.2)	5 (55.6)	0.33
**Stage**	I	2 (22.2)	0 (0)	
	II	5 (55.6)	7 (77.8)	
	III	2 (22.2)	2 (22.2)	0.6315
**Histological subtype**	Adenocarcinoma	7 (77.8)	8 (88.9)	
	Squamous cell carcinoma	2 (22.2)	1 (11.1)	1.0
**ECOG PS ^a^**	0	4 (44.4)	3 (33.3)	
	1	5 (55.6)	6 (66.7)	1.0
**Adjuvant treatment**	Yes	6 (66.7)	6 (66.7)	
	No	3 (33.3)	3 (33.3)	0.6171
**Type of surgery**	Lobectomy/bilobectomy	4 (44.4)	8 (88.9)	
	Pneumonectomy	3 (33.3)	1 (11.1)	
	Segmentectomy	2 (22.2)	0 (0)	0.4543
**Smoking history**	Current smoker	8 (88.9)	2 (22.2)	
	Former smoker	1 (11.1)	4 (44.4)	
	Never a smoker	0 (0)	3 (33.3)	0.1615

^a^ ECOG PS, Eastern Cooperative Oncology Group performance status.

**Table 2:** miRNAs differentially expressed in EV pulmonary TDV from relapsed vs. non-relapsed patients ordered according to their level of expression in descending order. The average trimmed mean of M-values (TMM) normalization for each miRNA is indicated for relapsed and non-relapsed patients.

miRNA Name	Log Fold Change	*p*-Value	Average TMM in Relapsed Patients	Average TMM in Non-Relapsed Patients
**hsa-miR-203a-3p**	1.6505	0.0067	2575.19	822.31
**hsa-miR-335-5p**	−1.2313	0.0245	599.65	1403.15
**hsa-miR-34a-5p**	−1.6527	0.0259	150.19	468.98
**hsa-miR-141-3p**	−1.3132	0.0216	134.64	333.56
**hsa-miR-200c-3p**	−1.4133	0.0141	91.94	244.24
**hsa-miR-200a-3p**	−1.6506	0.0200	66.97	207.77
**hsa-miR-200b-3p**	−1.5803	0.0220	56.13	165.26
**hsa-miR-335-3p**	−1.9874	0.0029	34.92	134.76
**hsa-miR-224-5p**	−1.3333	0.0285	38.74	93.53
**hsa-miR-424-5p**	1.0711	0.0146	66.23	29.7
**hsa-miR-483-3p**	1.5310	0.0144	52.39	19.38
**hsa-miR-429**	−2.0576	0.0089	10.66	44.39
**hsa-miR-574-5p**	−2.0495	0.0038	8.68	35.4
**hsa-miR-10b-3p**	1.6994	0.0179	35.94	12.57
**hsa-miR-10a-3p**	−1.9308	0.0069	7.83	29.45
**hsa-miR-1268b**	−1.6110	0.0228	4.29	15.36
**hsa-miR-582-3p**	−1.9282	0.0211	2.77	12.93

**Table 3:** Main clinical characteristics of validation cohort (*n* = 70).

Characteristics	Subtypes	*n* (%)	TTR
**Sex**	Male	51 (72.9)	
	Female	19 (27.1)	0.927
**Age, years**	Mean age (range)	64 (32–79)	
	≤65	36 (51.4)	
	>65	34 (48.6)	0.862
**Stage**	I	38 (54.3)	
	II	25 (35.7)	
	III	7 (10)	0.007
**Lymph node involvement**	N0	53 (75.7)	
	N+	17 (24.3)	0.001
**Histological subtype**	Adenocarcinoma	49 (70)	
	Squamous cell carcinoma	17 (24.3)	
	Other	4 (5.7)	0.530
**ECOG PS ^a^**	0	24 (34.3)	
	1	44 (62.9)	
	2	2 (2.9)	0.018
**Adjuvant treatment**	Yes	24 (34.3)	
	No	46 (65.7)	0.044
**Relapse**	Yes	25 (35.7)	
	No	45 (64.3)	-
**Type of surgery**	Lobectomy/bilobectomy	51 (72.9)	
	Pneumonectomy	8 (11.4)	
	Atypical resection	6 (8.6)	
	Segmentectomy	5 (7.1)	0.811
**Smoking history**	Current smoker	34 (48.6)	
	Former smoker	31 (44.3)	
	Never a smoker	5 (7.1)	0.504

^a^ ECOG PS, Eastern Cooperative Oncology Group performance status.

**Table 4:** Cox multivariate analysis for TTR in the overall cohort.

Time to Relapse	Hazard Ratio (95%CI)	*p*
**Stage I assay**	**0.174 (0.056–0.543)**	**0.003**
ECOG PS 0	0.360 (0.068–1.912)	0.230
No adjuvant treatment	1.375 (0.407–4.646)	0.609
**TDV EV-miR-203a-3p**	**2.442 (1.126–5.294)**	**0.024**

## Data Availability

Normalized microRNA seq data (TPM) from the screening cohort with indications of the relapse statuses of the patients have been provided as a Supplementary Material (Table S1).

## Supplementary Materials

The following supporting information can be downloaded at: www.mdpi.com/article/10.3390/ijms23137138/s1.
