# Supplementary material for: Characterization of the MicroRNA Cargo of Extracellular Vesicles Isolated from a Pulmonary Tumor-Draining Vein Identifies miR-203a-3p as a Relapse Biomarker for Resected Non-Small Cell Lung Cancer
Source: Int J Mol Sci. 2022 Jun 27;23(13):7138. doi: 10.3390/ijms23137138 (PMC9266391; doi:10.3390/ijms23137138)
Supplement: Supplementary file 1 [file ijms-23-07138-s001.zip › Supplementary_Figures.pptx]

## Slide 1
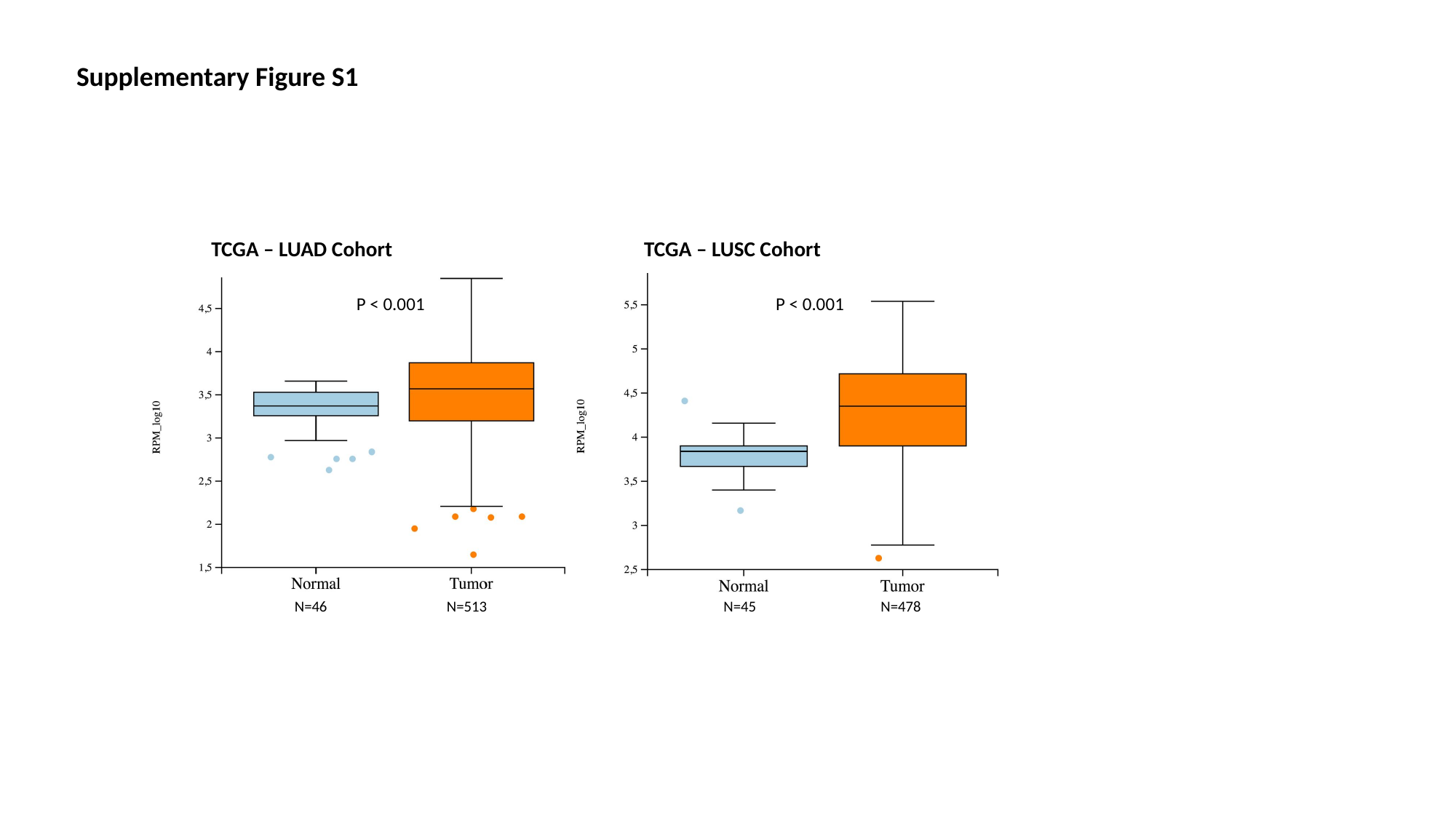

Supplementary Figure S1
TCGA – LUSC Cohort
TCGA – LUAD Cohort
P < 0.001
P < 0.001
N=46
N=513
N=45
N=478

## Slide 2
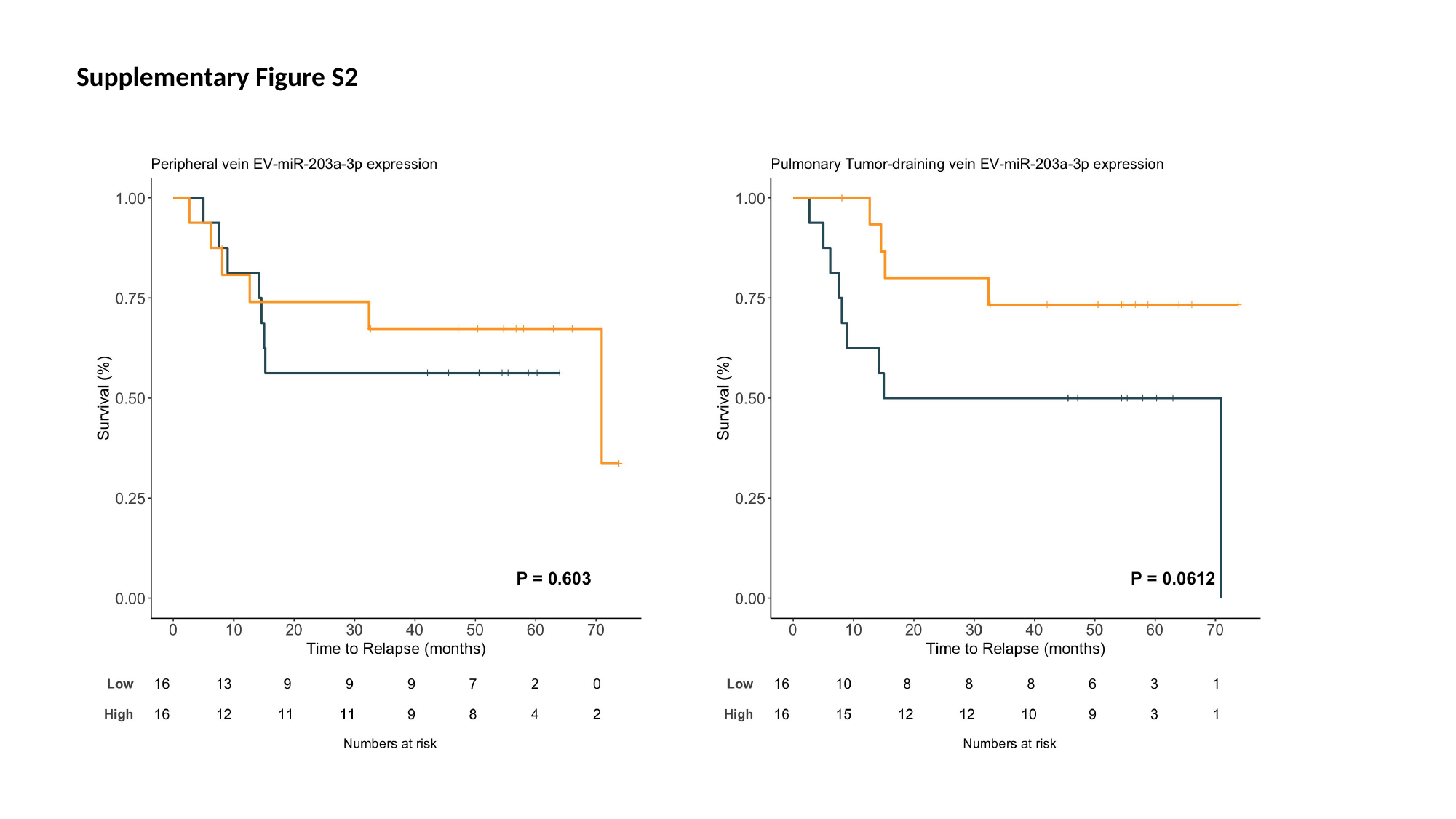

Supplementary Figure S2
